# Publisher Correction: A brainstem map for visceral sensations

**DOI:** 10.1038/s41586-022-05414-5

**Published:** 2022-10-14

**Authors:** Chen Ran, Jack C. Boettcher, Judith A. Kaye, Catherine E. Gallori, Stephen D. Liberles

**Affiliations:** Department of Cell Biology, Howard Hughes Medical Institute, Harvard Medical School, Boston, MA USA

**Keywords:** Sensory processing, Autonomic nervous system

Correction to: *Nature* 10.1038/s41586-022-05139-5 Published online 31 August 2022

In the version of this article initially published, there were errors in the second display equation in the Methods section, where the third, fourth and sixth closing brackets were missing. The equations have been corrected in the HTML and PDF versions of the article

