# Supplementary material for: A brainstem map for visceral sensations
Source: Nature. 2022 Aug 31;609(7926):320–6. doi: 10.1038/s41586-022-05139-5 (PMC9452305; doi:10.1038/s41586-022-05139-5)
Supplement: Supplementary file 1 — Reporting Summary [file 41586_2022_5139_MOESM1_ESM.pdf]

nature portfolio

Corresponding author(s): Stephen D. Liberles

Last updated by author(s): Jun 16, 2022

Reporting Summary

Nature Portfolio wishes to improve the reproducibility of the work that we publish. This form provides structure for consistency and transparency in reporting. For further information on Nature Portfolio policies, see our Editorial Policies and the Editorial Policy Checklist.

Statistics

For all statistical analyses, confirm that the following items are present in the figure legend, table legend, main text, or Methods section.

n/a	Confirmed	
☐	☒	The exact sample size (n) for each experimental group/condition, given as a discrete number and unit of measurement
☐	☒	A statement on whether measurements were taken from distinct samples or whether the same sample was measured repeatedly
☐	☒	The statistical test(s) used AND whether they are one- or two-sided<br>*Only common tests should be described solely by name; describe more complex techniques in the Methods section.*
☒	☐	A description of all covariates tested
☐	☒	A description of any assumptions or corrections, such as tests of normality and adjustment for multiple comparisons
☐	☒	A full description of the statistical parameters including central tendency (e.g. means) or other basic estimates (e.g. regression coefficient) AND variation (e.g. standard deviation) or associated estimates of uncertainty (e.g. confidence intervals)
☐	☒	For null hypothesis testing, the test statistic (e.g. F, t, r) with confidence intervals, effect sizes, degrees of freedom and P value noted<br>*Give P values as exact values whenever suitable.*
☒	☐	For Bayesian analysis, information on the choice of priors and Markov chain Monte Carlo settings
☒	☐	For hierarchical and complex designs, identification of the appropriate level for tests and full reporting of outcomes
☐	☒	Estimates of effect sizes (e.g. Cohen's d, Pearson's r), indicating how they were calculated

Our web collection on statistics for biologists contains articles on many of the points above.

Software and code

Policy information about availability of computer code

Data collection	Olympus FluoView software was used for collecting two-photon images.
Data analysis	Seurat (4.0.5), R (4.1.1), ImageJ (1.52q), Matlab (R2020a), Processing (4.0a3), Python (3.9.4), OpenCV (4.5.1), and GraphPad Prism (9.0.2) were used for for data analysis.

For manuscripts utilizing custom algorithms or software that are central to the research but not yet described in published literature, software must be made available to editors and reviewers. We strongly encourage code deposition in a community repository (e.g. GitHub). See the Nature Portfolio guidelines for submitting code & software for further information.

Data

Policy information about availability of data

All manuscripts must include a data availability statement. This statement should provide the following information, where applicable:

- Accession codes, unique identifiers, or web links for publicly available datasets
- A description of any restrictions on data availability
- For clinical datasets or third party data, please ensure that the statement adheres to our policy

Source data are provided with this paper at Nature, and raw images are available upon reasonable request.

Field-specific reporting

Please select the one below that is the best fit for your research. If you are not sure, read the appropriate sections before making your selection.

☒ Life sciences ☐ Behavioural & social sciences ☐ Ecological, evolutionary & environmental sciences

For a reference copy of the document with all sections, see nature.com/documents/nr-reporting-summary-flat.pdf

Life sciences study design

All studies must disclose on these points even when the disclosure is negative.

Sample size	Sample sizes were based on prior expertise and publications in our field, including Williams et al., Cell 2016, Zhao et al., Nature, 2022, Ichiki et al., Nature, 2022, and are disclosed in each figure legend.
Data exclusions	The following criteria were established prior to analysis: in experiments involving duodenal glucose, fields of view (FOVs) with at least five neurons responsive to every stimulus were used for further analysis; in all other experiments, FOVs with at least two responsive neurons to all stimuli analyzed were used. For spatial analysis of stimulus pairs, only FOVs containing at least 2 neurons selectively tuned to each stimulus were used for further analysis. Neuronal responses were all manually examined, and rarely (2.94%), responses were excluded due to motion artifacts, dramatic baseline drifting that could not be corrected by detrending, or by physical obstruction of the light path by debris. For chemogenetic activation experiment, 9 experimental mice were injected with clozapine-N-oxide. 1 mouse (out of 9) that did not display CNO-induced apnea was excluded.
Replication	All experiments were successfully reproduced. Extended Data Fig. 2a, b were reproduced with another set of the same stimulus in the same animal, and Extended Data Fig. 7a was reproduced in three animals in total. The numbers of animals used in all figure were reported in figure legends in detail.
Randomization	No method of randomization was used to determine how animals were allocated to experimental groups in Fig. 3, and all other analyses involved stimulus comparisons in the same animals.
Blinding	We did not perform the analyses blinded except for Extended Data Fig. 8, in which GFP-labeled boutons were determined by an experimenter blind to neuronal response properties or image orientation, and areas of innervation were determined manually by an experimenter blind to neuronal responses and tracer injection site (group allocation). Other experiments were analyzed using computer codes and were not subjected to potential experimenter bias.

Reporting for specific materials, systems and methods

We require information from authors about some types of materials, experimental systems and methods used in many studies. Here, indicate whether each material, system or method listed is relevant to your study. If you are not sure if a list item applies to your research, read the appropriate section before selecting a response.

Materials & experimental systems

n/a	Involved in the study	
☒	☐	Antibodies
☒	☐	Eukaryotic cell lines
☒	☐	Palaeontology and archaeology
☐	☒	Animals and other organisms
☒	☐	Human research participants
☒	☐	Clinical data
☒	☐	Dual use research of concern

Methods

n/a	Involved in the study	
☒	☐	ChIP-seq
☒	☐	Flow cytometry
☒	☐	MRI-based neuroimaging

Animals and other organisms

Policy information about studies involving animals; ARRIVE guidelines recommended for reporting animal research

Laboratory animals	Animals were maintained under constant temperature (23 ± 1°C) and relative humidity (46 ± 5%) with a 12-h light/dark cycle. Mice used in this study are from both sexes and of a mixed genetic background. Glp1r-ires-Cre (029283), Gpr65-ires-Cre (029282) and Crhr2-ires-Cre (033728) mice were made previously in the lab and are now available at Jackson Laboratory. Slc17a6-ires-Cre (Vglut2-ires-Cre, 028863), Slc32a1-ires-Cre (Vgat-ires-Cre, 028862), Rosa26-lsl-Gfp-L10a (024750), Tac1-ires-Cre (021877), Pdyn-ires-Cre (027958), Penk-ires-Cre (025112), Cartpt-ires-Cre (028533), Sst-ires-Cre (013044), Th-Cre (021877), and Gcg-Cre (030542) mice were purchased from Jackson Laboratory. All mice used in the experiments were older than 2 weeks.
Wild animals	No wild animals were used.
Field-collected samples	No field-collected samples were used.

Ethics oversight	All animal procedures followed the ethical guidelines outlined in the NIH Guide for the Care and Use of Laboratory Animals, and all protocols were approved by the institutional animal care and use committee (IACUC) at Harvard Medical School.

Note that full information on the approval of the study protocol must also be provided in the manuscript.
